# Seed Functional Traits Provide Support for Ecological Restoration and *ex situ* Conservation in the Threatened Amazon Ironstone Outcrop Flora

**DOI:** 10.3389/fpls.2020.599496

**Published:** 2020-12-08

**Authors:** Marcilio Zanetti, Roberta L. C. Dayrell, Mariana V. Wardil, Alexandre Damasceno, Tais Fernandes, Alexandre Castilho, Fernando M. G. Santos, Fernando A. O. Silveira

**Affiliations:** ^1^Bioma meio Ambiente LTDA, Nova Lima, Brazil; ^2^Departamento de Genética, Ecologia e Evolução, Universidade Federal de Minas Gerais, Belo Horizonte, Brazil; ^3^School of Biological Sciences, University of Western Australia (UWA), Perth, WA, Australia; ^4^Amplo Engenharia e Gestão de Projetos LTDA, Belo Horizonte, Brazil; ^5^VALE S/A. Environmental Licensing Management, Mina de Águas Claras, Nova Lima, Brazil

**Keywords:** Carajás, conservation, dormancy, functional traits, germination requirements, phylogeny, seed quality

## Abstract

Cangas (ironstone outcrops) host a specialized flora, characterized by high degree of edaphic endemism and an apparent lack of natural history knowledge of its flora. Due to intense pressure from iron ore mining this ecosystem is under threat and in need of restoration. We studied seed functional traits that are relevant for restoration, translocation and *ex situ* conservation in 48 species from cangas in eastern Amazon. Were determined the thermal niche breadth, classified seed dormancy and determined methods to overcome it, determined the effect of seed storage on germination, tested the association between germination traits and functional groups, and tested whether seed traits are phylogenetically conserved. We found a broad interspecific variation in most seed traits, except for seed water content. Large interspecific variation in the temperature niche breadth was found among the studied species, but only four species, showed optimum germination at high temperatures of 35–40°C, despite high temperatures under natural conditions. Only 35% of the studied species produced dormant seeds. Mechanical scarification was effective in overcoming physical dormancy and application of gibberellic acid was effective in overcoming physiological dormancy in five species. For the 29 species that seeds were stored for 24 months, 76% showed decreases in the germination percentage. The weak association between germination traits and life-history traits indicate that no particular plant functional type requires specific methods for seed-based translocations. Exceptions were the lianas which showed relatively larger seeds compared to the other growth-forms. Dormancy was the only trait strongly related to phylogeny, suggesting that phylogenetic relatedness may not be a good predictor of regeneration from seeds in cangas. Our study provides support to better manage seed sourcing, use, storage and enhancement techniques with expected reduced costs and increased seedling establishment success.

## Introduction

*Ex situ* conservation and ecological restoration are examples of parallel strategies that have been increasingly advocated as important elements in conservation of biodiversity at both regional and global scales (Li and Pritchard, 2009; Sharrock et al., 2014; O’Donnell and Sharrock, 2017; Wyse et al., 2018). The successful implementation of cost-effective conservation strategies requires solid knowledge on seed biology, including the identification of appropriate techniques of seed collection, genetic diversity issues, determination of physiological requirements for seed germination, storage behavior, and dormancy classification and alleviation (FAO, 1993; Hamilton, 2001; Broadhurst et al., 2008; Breed et al., 2012; Espeland et al., 2016; Miller et al., 2017).

Seeds play a critical role in *ex situ* conservation and restoration ecology (Miller et al., 2017; Kildisheva et al., 2020). Understanding germination requirements is a basic step to determine suitable conditions for translocation (Commander et al., 2018) and optimize seed sowing and seedling establishment, for example, through dormancy alleviation strategies (Turner et al., 2013). The determination of the percentage of dormant, inviable, viable and empty seeds provides important information for estimating and calculating the number of seeds to be used in ecological restoration and *ex situ* conservation programs (Erickson et al., 2016, 2017; Kildisheva et al., 2020). These traits are particularly appropriate for informing species selection (Pywell et al., 2003), planning methods of species propagation and designing seed sowing strategies (Miller et al., 2017). For example, knowledge on interspecific variation in mean germination time can be used to accelerate soil cover and outcompete invasive species (Rowe and Leger, 2011; Gioria et al., 2016). In turn, species with slower germination may be sowed during latter stages of regeneration or in invasive-free sites.

Germination niche breadth can be an important driver of species distribution range, adult niche breadth and microhabitat segregation (Donohue et al., 2010; Fernández-Pascual et al., 2013; Marques et al., 2014). The correct identification of temperature requirements for germination is important for the determination of microclimatic conditions when selecting sites for translocation programs (Commander et al., 2018). Data from basic seed germination experiments also can be used in association with seed enhancement technologies (Pedrini et al., 2020) by industry to identify beneficial techniques performed on seeds after harvest, adding “value” on a given seed lot, improving its germination rates and seedling survival (Taylor et al., 1998; Madsen et al., 2016; Balazs et al., 2020; Frischie et al., 2020), and likelihood of establishment in restoration projects. Finally, determining the patterns of *ex situ* seed storage is key to determine seed usage guidelines, optimize biological material and economic resources (Miller et al., 2017; de Vitis et al., 2020).

Our focus here was on the endemic and threatened flora growing on ironstone outcrops (banded iron formations locally known as *cangas*) which are extensively explored for open cast iron mining (Viana et al., 2016; Mota et al., 2018). Owing to a high degree of endemism and newly discovered species (Viana et al., 2016; Mota et al., 2018; Giulietti et al., 2019), knowledge gaps on the ecology of ironstone endemics need to be filled to implement appropriate conservation and management programs (Zappi et al., 2019). We report the first community-wide study on germination, dormancy and storage including 48 species from the eastern Amazon. The ecology of plants growing on ironstone outcrops is virtually unknown. Despite recent studies addressing the restoration of open cast mining, we still lack a broad understanding on basic aspects of seed ecology of ironstone outcrop endemics because species prioritization studies have overlooked seed functional traits (e.g., Giannini et al., 2016; Zappi et al., 2018; Gastauer et al., 2020, but see Ramos et al., 2019).

Our goal was to explore the diversity of seed functional traits in 48 species under the influence of mining activity to provide restoration-relevant data that can support better informed-decisions on translocation, restoration and *ex situ* conservation strategies. Specifically, we aimed at: (1) determining germination thermal niche breadth; (2) screening species for the presence of dormancy and methods to alleviate it; (3) determining the effect of seed storage on germination to determine seed storage capacity in room temperature and its subsequent use in conservation programs; (4) test the association between germination traits and functional groups (life-history, dormancy class, growth-form and dispersal period) to explore the putative drivers of the ecology and evolution of germination traits (Donohue et al., 2010; Escobar et al., 2018); and (5) test whether seed traits can be predicted by phylogenetic affinity, which can allow us to predict seed functional traits in unassessed taxa, and to improve our understanding on germination patterns across communities (Dayrell et al., 2017).

## Materials and Methods

### Study Site, Study Species and Seed Collection

**FIGURE 1:**
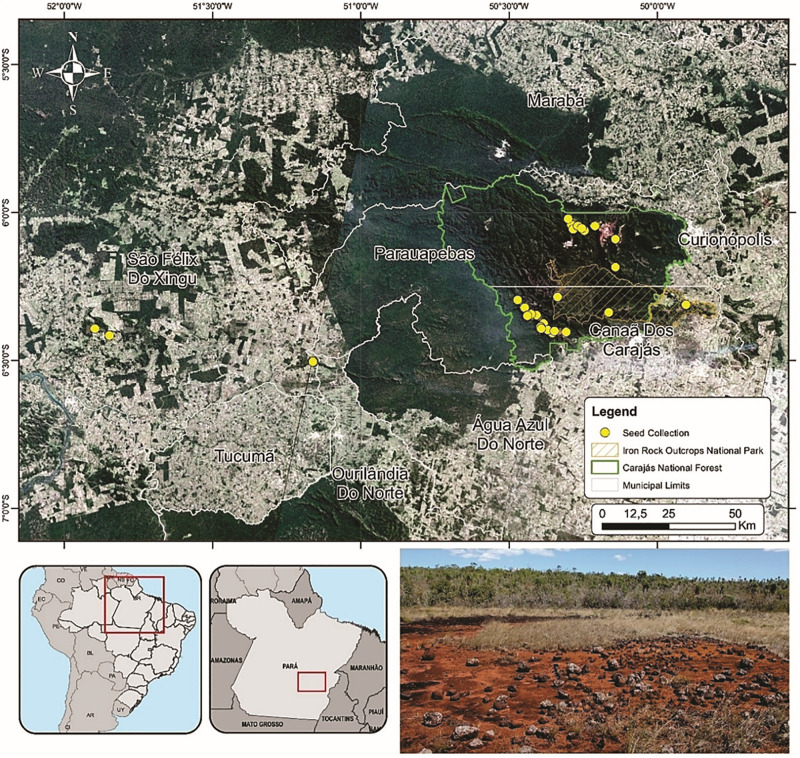
Map of the Carajás National Forest where seeds of 48 species were sampled (yellow dots). The bottom right panel shows a typical ironstone outcrop landscape during the dry season.

We sampled seeds from species growing directly on ironstone outcrops which harbor a large proportion of endemic and threatened species due to mining activities (Viana et al., 2016; Mota et al., 2018). Field work was carried out in the Carajás National Forest, Campos Ferruginosos National Park and on the surrounding plateaus, including the Bocaina, Cristalino, Serra de Campos and Serra Arqueada mountain ranges in Pará, North Brazil (Figure 1). All sites are located in the eastern Amazon, which is characterized by the presences of isolated, naturally fragmented highlands surrounded by a matrix of lowland tropical rainforest (Figure 1). The summits of these table mountains are formed by ferruginous iron-rich rock outcrops covered by a mosaic of open shrublands, heathlands and grasslands (Viana et al., 2016). Soil depth plays a key role in shaping physiognomies (Schaefer et al., 2009) where many species grow directly on ironstone outcrops or on nutrient-poor, metal-rich (Nunes et al., 2015), usually shallow pteric plinthsols and cambisols, latosols and organosols (Schaefer et al., 2007).

The hot and humid tropical climate falls into the “Aw” classification of Köppen (Alvares et al., 2013), with a marked dry season from April to September and a rainy season from October to March (Giannini et al., 2016). The total annual precipitation ranges from 1,800 to 2,300 mm, with an average of 1,550 mm during the rainy season, and 350 mm during the dry season (Moraes et al., 2005). The average monthly temperatures range from 19 to 31°C with a mean annual temperature of around 26°C (Instituto Brasileiro do Meio Ambiente e dos Recursos Naturais Renováveis – IBAMA, 2003; Moraes et al., 2005). Soil surface temperatures may reach up to 70°C (Carmo et al., 2012). Currently, iron ore production at Serra dos Carajás has had profound social-economic impacts at the local and regional scales and accounts for about 30% of the national production (Tolbert et al., 1971; Viana et al., 2016; Sonter et al., 2017). At the study site, open cast mining relies on whole topsoil removal for mineral extraction, resulting in degraded, unvegetated substrates.

The recently concluded Flora of the cangas of Carajas registered 856 angiosperms from the Carajas National Forest and Campos Ferruginosos National Park. Our focus here was on the endemic and threatened flora growing on ironstone outcrops (banded iron formations locally known as cangas) which are extensively explored for open cast iron mining (Viana et al., 2016; Mota et al., 2018). Owing to a high degree of endemism and newly discovered species (Viana et al., 2016; Mota et al., 2018; Giulietti et al., 2019), knowledge gaps on the ecology of ironstone endemics need to be filled to implement appropriate conservation and management programs (Zappi et al., 2019). Giulietti et al. (2019) concluded that 38 of the 856 species registered by Mota et al. (2018) are edaphic endemics restricted from the cangas. We report the first community-wide study on germination, dormancy and storage including 48 species from the eastern Amazon (5% of the 856 spp.), and 20 out of 38 endemics (55.2%. Four species are threatened in the Brazilian National threatened plant species list and, two threatened species not present in the list of edaphic endemic species and four locally rare species not restricted from the canga soils (*Praxelis asperulaceae*, *Chelonanthus purpurascens*, *Ruellia anamariae* and *Cavalcatia percymosa*). The ecology of plants growing on ironstone outcrops is virtually unknown. Despite recent studies addressing the restoration of open cast mining, we still lack a broad understanding on basic aspects of seed ecology of ironstone outcrop endemics because species prioritization studies have overlooked seed functional traits (e.g., Giannini et al., 2016; Zappi et al., 2018; Gastauer et al., 2020, but see Ramos et al., 2019).

We collected seeds from 48 species distributed in 36 genera and 19 families (Table 1). Twenty-one species (43.7%) are endemic to Carajás, and among these, four are highly restricted endemics and nine are ranged restricted endemic (Giulietti et al., 2019). Six species are threatened (*M. carajensis* and *M. skinneri* var. *carajarum* are critically endangered, and *I. cavalcantei, H. paraense, A. carajasensis* and *P. microphyllus* endangered) (Ministério do Meio Ambiente (MMA), 2014; Table 1).

**TABLE 1:** List of species, threatened category, geographic distribution (HRE = highly restricted endemic and RRE = ranged restricted endemic), growth-form, life-history, storage experiment (X denotes species with enough seeds to run the experiments), dormancy (ND = non-dormant, PY = physically dormant seeds, PD = physiologically dormant seeds), percentage of initial seed viability (ISV%), percentage of seed water content (SWC%), percentage of embryoless seeds and dispersal season of 48 species sampled in ironstone outcrops in Carajás, eastern Amazon.

**Family**	**Species**	**Conservation**	**Distribution**	**Growth-form**	**Life history**	**Storage**	**Dormancy**	**ISV (%)**	**SWC (%)**	**Embryoless (%)**	**Dispersal season**
Asteraceae	*Cavalcantia glomerata R.M.King& H.Rob.*	NE	Carajás RRE	Herb	Annual	X	PD	63.7	9.4	1	Dry
	*Cavalcantia percymosa*	NE	South Pará	Herb	Annual	X	PD	82.2	4.5	2	Dry
	*Lepidaploa arenaria*	NE	Brazil	Shrub	Annual	X		34	8.9	66	Dry
	*Lepidaploa paraensis*	NE	Carajás RRE	Shrub	Perennial	X		25	7.6	93	Dry
	*Monogereion carajensis*	CR	Carajás	Herb	Annual		PD	60.5	14.2	0	Dry
	*Parapiqueria cavalcantei*	NE	Carajás HRE	Herb	Annual	X		89.1	9.8	16	Dry
	*Praxelis asperulacea*	NE	South America	Herb	Annual	X		85.7	9.1	21	Dry
Bignoniaceae	*Anemopaegma carajasense*	–	Carajás RRE	Shrub	Perennial	X		93	7.2	0	Dry
Bromeliaceae	*Dyckia duckei*	NE	Brazil	Herb	Perennial			82.6	13.5	35	Dry
Convolvulaceae	*Ipomoea cavalcantei*	EN	Carajás HRE	Liana	Perennial			48.6	11.3	0	Dry
	*Ipomoea marabaensis*	NE	North Brazil	Liana	Perennial		PY	12	10.3	0	Dry
	*Ipomoea maurandioides*	NE	South America	Liana	Perennial		PY	77.3	7.8	0	Dry
	*Operculina hamiltonii*	NE	Americas	Liana	Perennial		PY	66	6	0	Dry-rainy
Cyperaceae	*Bulbostylis cangae*	–	Carajás RRE	Herb	Perennial	X	PD	63.8	7.7	12	Rainy transition
	*Bulbostylis conifera*	NE	South America	Herb	Annual		PD	54.6	4.6	8	Dry
	*Hypolytrum paraense*	EN	Pará	Herb	Perennial	X	PD	68.6	14.8	0	Rainy
Eriocaulaceae	*Paepalanthus fasciculoides*	NE	South America	Herb	Annual	X		88	3.9	0	Dry
Fabaceae	*Bauhinia pulchella*.	NE	South America	Shrub	Perennial	X		84	15.9	3	Dry
	*Chamaecrista desvauxii*	NE	Americas	Shrub	Perennial	X		53.7	8.9	8	Dry
	*Dioclea apurensis*	NE	South America	Liana	Perennial		PY	68	10	5	Dry
	*Mimosa acutistipula* var. *ferrea*	NE	Brazil	Shrub	Perennial	X		86.6	10.7	0	Dry
	*Mimosa dasilvae*	NE	Carajás HRE	Shrub	Perennial	X		32.6	11.8	0	Dry
	*Mimosa skinneri* var. *carajarum*	CR	Carajás	Herb	Perennial		PY	78	7.5	0	Dry
Gentianaeceae	*Chelonanthus purpurascens*	NE	South America	Herb	Annual	X		85.2	10.2	4	Rainy-dry transition
Lentibulariaceae	*Utricularia neottioides*	NE	South America	Herb	Annual		PD	12.4	23	3	Dry
	*Utricularia physoceras*	NE	Carajás RRE	Herb	Annual		PD	7.1	25	7	Dry
	*Utricularia subulata*	NE	Pantropical	Herb	Annual		PD	5.8	–	3	Rainy-dry transition
Melastomataceae	*Brasilianthus carajensis*	NE	Carajás	Herb	Annual	X		85.3	6.2	23	Dry
	*Pleroma carajasense*	-	Carajás	Shrub	Perennial			63	–	41	Dry
	*Tibouchina edmundoi*	NE	North Brazil	Shrub	Perennial	X		53.3	12.1	98	Dry
Ochnaceae	*Sauvagesia tenella*	NE	Americas	Herb	Annual		PD	33.8	5.5	5	Rainy-dry transition
Orchidaceae	*Epidendrum nocturnum*	NE	Americas	Herb	Perennial	X		43	21.7	0	Dry
	*Sobralia liliastrum*	NE	South America	Herb	Perennial	X		19	22.5	0	Dry
Orobanchaceae	*Buchnera carajasensis*	–	Carajás	Herb	Annual		PD	38.3	4.8	7	Dry
Piperaceae	*Peperomia* sp.	–	–	Herb	Annual	X		85	4.3	5	Dry
Poaceae	*Axonopus carajasensis*	EN	Carajás RRE	Herb	Perennial			30	9.99	83	Rainy-dry transition
	*Axonopus longispicus*	NE	South America	Herb	Perennial	X		34.6	11.1	84	Rainy
	*Paspalum cangarum*	NE	Carajás RRE	Herb	Perennial	X		33.3	10.3	90	Rainy-dry transition
	*Paspalum cinerascens*	NE	South America	Herb	Perennial	X		17.6	10.8	97	Rainy-dry transition
	*Sporobolus multiramosus*	NE	Carajás RRE	Herb	Annual	X		69.7	12.4	1	Dry
	*Trachypogon spicatus*	NE	Widespread	Herb	Perennial	X		50	7.7	82	Rainy-dry transition
Rubiaceae	*Borreria elaiosulcata*	NE	Carajás RRE	Shrub	Annual	X		80	11.5	4	Dry
	*Borreria carajasensis*	NE	Carajás	Shrub	Annual	X		87.8	10.4	4	Dry
	*Carajasia cangae*	NE	Carajás HRE	Herb	Perennial	X	PD	38.1	13	4	Dry
	*Mitracarpus carajasensis*	NE	Carajás	Herb	Annual	X		86	12.7	0	Dry
Rutaceae	*Pilocarpus microphyllus*	EN	Widespread	Shrub	Perennial			58	7.7	0	Dry
Velloziaceae	*Vellozia glauca*	NE	Brazil	Shrub	Perennial	X		99.3	26.4	18	Dry
Xyridaceae	*Xyris brachysepala*	NE	Carajás	Herb	Annual	X		85.3	10.8	0	Dry

*NE = Not classified as threat level; EN = Endangered; CR = Critically endangered (According to the latest version of the Brazilian Red Book of Flora of 2013 and the Official National List of Threatened Flora Species of 2014). Southern Pará endemics: All iron rock plateau in the Southeastern Pará region. Carajás endemics: Restricted to the mineral province of Carajas (North, South and East ranges + National Park); As a criterion for the distribution classification, the flora of Brazil, the Carajás floras and species description articles were used.*

To cover all fruiting seasons, field trips were carried out monthly over a period of ten months, from March to December 2017. Monthly collections were carried out for ten days per month, with a total effort of 100 days of seed collection. To increase genetic diversity in our sampling, fruits were collected from as many individuals as possible for each species, with care to avoid compromising the viability of populations of the species sampled (Nevill et al., 2018). As the studied species have different distribution patterns, life histories and population sizes, including restricted range endemics and long-range endemics distributed beyond the studied area, seed collections for each species varied between the studied species. In general, wide range endemics were collected from more than one location and restricted range endemics were collected from only one location. To guarantee the best maturation stage of seeds and higher number of seeds collected, species flowering and fruiting ripening were monitored. Additional data about populations collections are included in Supplementary Table 1.

We collected seeds from 48 species distributed in 36 genera and 19 families (Table 1). Twenty species (41.6%) are endemic to Carajás, and among these, ten are highly restricted endemics (Giulietti et al., 2019). Six species are threatened (*Monogereion carajensis* and *Mimosa skinneri* var. *carajarum* are critically endangered, and *I. cavalcantei, H. paraense, A. carajasensis* and *P. microphyllus* endangered) (Brazil MMA 2014; Table 1).

### Germination and Thermal Niche Experiments

Fresh, externally apparently healthy seeds were subjected to experiments within 2 weeks after collection. To determine the regenerative potential from seeds, we classified seeds in four categories: viable, embryoless, non-viable and dormant (*sensu* Baskin and Baskin (2014). This classification system consists of a hierarchical classification (Dayrell et al., 2017), and allows distinguishing seeds that cannot germinate because embryos are lacking, are unviable or seeds required dormancy-alleviation treatments. More importantly, this system shows that the total of potentially germinable seeds in restoration-relevant activities should exclude embryoless seeds and seeds with non-viable embryos (see also Kildisheva et al., 2020).

To determine the percentage of embryoless seeds, four replicates of 25 seeds for each species were opened under a stereoscope to determine embryo presence. To determine embryo viability, the tetrazolium test was performed. For this test, we gently cut a small piece of the seed coat for species we suspected that had physical dormancy. Then, seeds were submitted to imbibition in distilled water for 24 h and then cut with a magnifying glass, immersed in 1% tetrazolium solution for 24 h and incubated in a germination chamber under a constant temperature of 25°C in the dark. Red stained embryos were considered viable and uncolored embryos were considered non-viable (Delouche et al., 1962).

To determine seed water content, four replicates of 25 fresh seeds per species were weighed on a digital scale, oven dried at 70°C for five days and reweighed. Seed water content (SWC) was then calculated according to the equation:

$$S\,W\,C=\frac{F\,M-D\,M}{D\,M}\times 100$$

where FM equals fresh mass and DM equals dry mass.

Before the germination tests, seed coats were sterilized with 1% sodium hypochlorite solution for 5 min and washed in tap water for 30 min to reduce the likelihood of fungal infestation. Seeds were then placed in Petri dishes (six replicates of 25 seeds in each temperature), lined with a double sheet of filter paper and moistened with 3 ml of 0.1M Nistatina to prevent further fungi growth. The Petri dishes were placed into germination chambers under a 12-h photoperiod and constant temperatures of 20, 25, 30, 35 and 40°C). These temperatures were chosen in an attempt to simulate the soil temperatures at the study site using data captured by data logger HOBO pendant mx2202. Soil temperatures ranged from 17.8 to 57.5°C with an average of 28.8°C (unpublished data). For ten species, we were not able to expose seeds to the thermogradient due to seed shortage. For *Ipomoea cavalcantei*, *Ipomoea marabaensis*, *Pilocarpus microphyllus*, *Sauvagesia tenella* e *Bulbostylis conifera* the experiments were set up in just one temperature, for *Utricularia subulata* in just two temperatures, for *Pleroma carajasense* and *Dioclea apurensis* in just three temperatures and for *Axonopus carajasensis* and *Operculina hamiltonii* experiments were set up at four temperatures. For these species, we assumed that 25°C is the optimum temperature for germination.

Germination was checked every 24 h and the criterion to consider a seed germinated was radicle protrusion (Bewley et al., 2013). After 50 days, the seeds that did not germinate were submitted to the tetrazolium test to examine embryo viability and to support dormancy classification (Baskin and Baskin, 2014). The optimum temperature was estimated by observing the temperature that the species presented the highest percentage of germination in the shortest mean time. The mean germination time, in days, was calculated following Ranal and Santana (2006):

$$M\,G\,T=\frac{\sum N_{i}\,T_{i}}{N_{i}}$$

where Ni is the number of seeds germinated on day i, and Ti is time i.

### Seed Storage Effect on Germination

We stored seeds of 29 species that had enough seeds available, with the aim of studying the effect of storage on germination. The seeds were stored at room temperature for 24 months in sealed ziplock bags or in Eppendorf tubes, wrapped in aluminum foil to protect against photodegradation. The ambient air temperature during the 24 months of storage, ranged from 7.7 to 36.1°C with an average of 21.9°C, and the average daily relative air humidity ranged from 30.9 to 91.2% with an average of 65.3% (National Meteorological Institute (INMET), 2020). After storage, the seeds were submitted to germination experiments at the optimum temperature and under the same conditions and experimental setting of the fresh seeds.

### Dormancy Classification and Alleviation

We used Baskin and Baskin (2014) to classify seed dormancy. The assessment of dormancy classification can be done through conducting a series of experimental steps to determine seed morphological and physiological traits. The first step is to determine whether the seeds germinate over a wide range of environmental conditions. Seeds with viable embryos and a low percentage of germination across the temperature gradient were classified as dormant. To assign dormancy classes, fresh seeds were submitted to imbibition tests to determine seed coat permeability. Imbibition tests consist of weighing the fresh mass of four replicates of 25 seeds each and weighing them after 72 h of immersion in distilled water (Kildisheva et al., 2020). Twenty-five seeds were half-cut to allow examination of embryo development. Seeds germinating within 30 days were scored as non-dormant. Seeds with permeable coats and well-developed embryos not germinating within four weeks were classified as physiologically dormant. Seeds with impermeable coats did not imbibe and were classified as physically dormant if germinated after scarification (Baskin and Baskin, 2014).

After classification, dormant seeds underwent dormancy-breaking experiments. For physically dormant seeds (PY), mechanical scarification was performed by comparing germination of control seeds vs seeds gently scarified with sandpaper. For physiologically dormant seeds (PD), germination tests with gibberellic acid (GA3, Sigma Aldrich) were performed in two concentrations: 125 and 250 μM, with the seeds submitted to imbibition for 48 h. Seeds not exposed to gibberellin were the control group. For each treatment, four replicates of 25 seeds were placed in germination chambers under a constant temperature of 25°C in a 12-h photoperiod for 30 days, with germination checked every 24 h.

### Data Analyses

We tested the effects of temperature treatments on two response variables: final germination proportion and mean germination time (MGT). For germination proportion, we ran a generalized linear model (GLM) with a logit link function specifying a binomial error structure. When the data were overdispersed, we used a quasi-binomial error distribution, which fitted better to the model in most cases. We analyzed MGT using a linear model. To determine statistically significant differences in germination proportion and mean germination time among temperatures, we performed Tukey’s post hoc test using the Multcomp package (Hothorn et al., 2008).

To compare the effect of dormancy-breaking treatments, we ran GLM with a logit link function and a binomial error structure. When the data were overdispersed, we used a quasi-binomial error distribution, which fitted better to the model in most cases. To determine significant statistical differences among treatments, we performed Tukey’s post hoc test using the Multcomp package (Hothorn et al., 2008).

We used the optimum temperature of germination to calculate the phylogenetic signal and the principal components analysis. The optimum temperature was corrected using only the initial number of viable seeds and the percentage of viable seeds was corrected using only the number of filled seeds. Temperature range of germination (T range) was used to calculated thermal niche breadth. T range was calculated by subtracting the highest temperature in which seed germination occurred by the lowest temperature in which germination occurred. Stenothermic species germinated in T ranges smaller than 10^°^C, whereas eurythermic species germinated in T ranges higher than 10^°^C.

Values of germination traits were transformed to provide distributions as close as possible to normality prior to the principal component analysis (PCA). Arcsin of the square root transformation was used for the germination percentage, viability and fruiting season, whereas log transformation was employed for seed mass, SWC and embryoless seeds percentage. No transformation improved distribution of T range. MGT did not require transformation. Few species lacked values for MGT, SWC and seed mass due to the absence of germination, and experimental constraints. We used the R package *missMDA* (Husson and Josse, 2018) to replace the missing values for the PCA and estimate parameters based on existing values within the dataset (Josse and Husson, 2012, 2016). A PCA including germination percentage, MGT, initial viability, embryoless percentage, seed mass, SWC and fruiting duration was performed using the “principal” function within the *psych* package (Revelle, 2017). Principal components one and two were plotted using “ggbiplot” and confidence ellipses were set to represent 95% intervals of confidence around the following groups: seed dispersal season (rainy, dry season or transition rainy-dry), dormancy (dormant or nondormant), temperature range (eurythermic or stenothermic), habit (herb, liana or shrub), and life history (annual or perennial).

For the seed storage experiment, data on germination percentage and MGT were tested for normality by the Kolmogorov-Smirnov test and homoscedasticity by the Barlett test. The data met the assumptions of parametric analyses and means were compared by paired T-tests. All data are presented as mean ± standard deviation. α values of 0.05 were used in all analyses.

To calculate the phylogenetic signal, the initial topology of the phylogenetic tree with the 48 sampled species was obtained using the Phylomatic (Webb and Donoghue, 2005). To improve node resolution and to obtain more precise estimates of branch lengths, estimated ages of 31 nodes were obtained in several studies (Supplementary Material 1). Thereafter, non-dated nodes were positioned evenly between dated nodes using bladj algorithm of Phylocom software (Webb et al., 2008). For quantitative traits we performed Blomberg’s K test with 100,000 randomizations (Blomberg et al., 2003; Münkemüller et al., 2012) in the *picante* package (Kembel et al., 2010). A K less than one implies that species resemble each other less than expected under Brownian motion evolution along the phylogenetic tree, whereas K values greater than one imply that close relatives are more similar than expected under Brownian motion evolution (Blomberg et al., 2003). We used the Maddison and Slatkin (1991) method to estimate the phylogenetic signal categorical traits. This method compares the minimum number of trait-state transitions across a phylogenetic tree with a null model (100,000 randomizations), in which the trait states were randomized in the tips of the tree. If related species have similar trait states, the number of evolutionary transitions observed will be lower than expected based on the null model (Maddison and Slatkin, 1991). Phylogenetic analyses were conducted using the R (R Core Team, 2018).

## Results

### Determining Germination Thermal Niche Breadth

**FIGURE 2:**
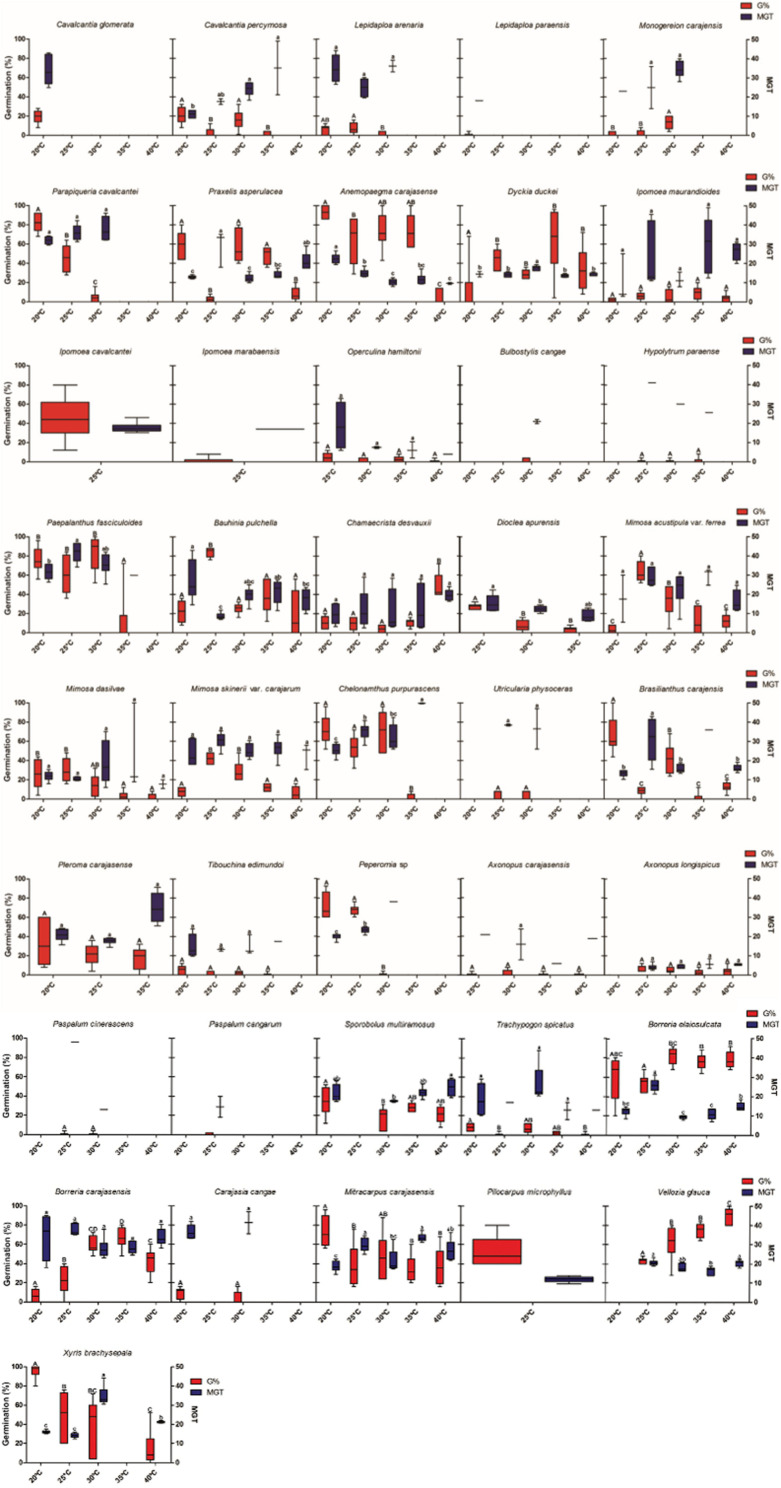
Thermal niche breadth and mean germination time of 48 species from ironstone outcrops in eastern Amazon. The boxplots present the germination values and mean germination time, showing maximum, minimum and average values. G% = Percentage of germination (red boxes); MGT = Average germination time (days, blue boxes).

Thermal niche breadth was variable across species (Figure 2). Thirteen species were classified as stenothermic with germination observed only under one, two or three temperatures. However, 23 species were eurythermics, germinating under four or five temperatures, including *Axonopus carajasensis* and *Operculina hamiltonii*, which had their experiments set up at just four temperatures and showed germination at all tested temperatures. Four species (*Utricularia neottioides*, *Epidendrum nocturnum*, *Sobralia liliastrum* and *Buchnera carajasensis*) did not germinate at any temperature, and eight species (*Ipomoea cavalcantei, Ipomoea marabaensis*, *Pilocarpus microphyllus, Dioclea apurensis, Pleroma carajasense*, *Utricularia subulata*, *Sauvagesia tenella* and *Bulbostylis conifera*) have not been classified due to seed shortage.

Optimum temperature for seed germination also showed a wide variation among species. Most species showed optimal temperatures between 20 and 30°C range, with only two species showing optimal temperatures at 35°C (*Dickia duckei* and *Ipomoea maurandioides*), and two at 40°C (*Chamaecrista desvauxii* and *Vellozia glauca*) (Figures 2A,B). MGT was 20.8 days on average, but was also variable among species. *Axonopus longispicus* showed the faster germination (8.2 days), and *Utricularia physoceras* the slowest (38.5 days).

### Dormancy Classification and Alleviation

**FIGURE 3:**
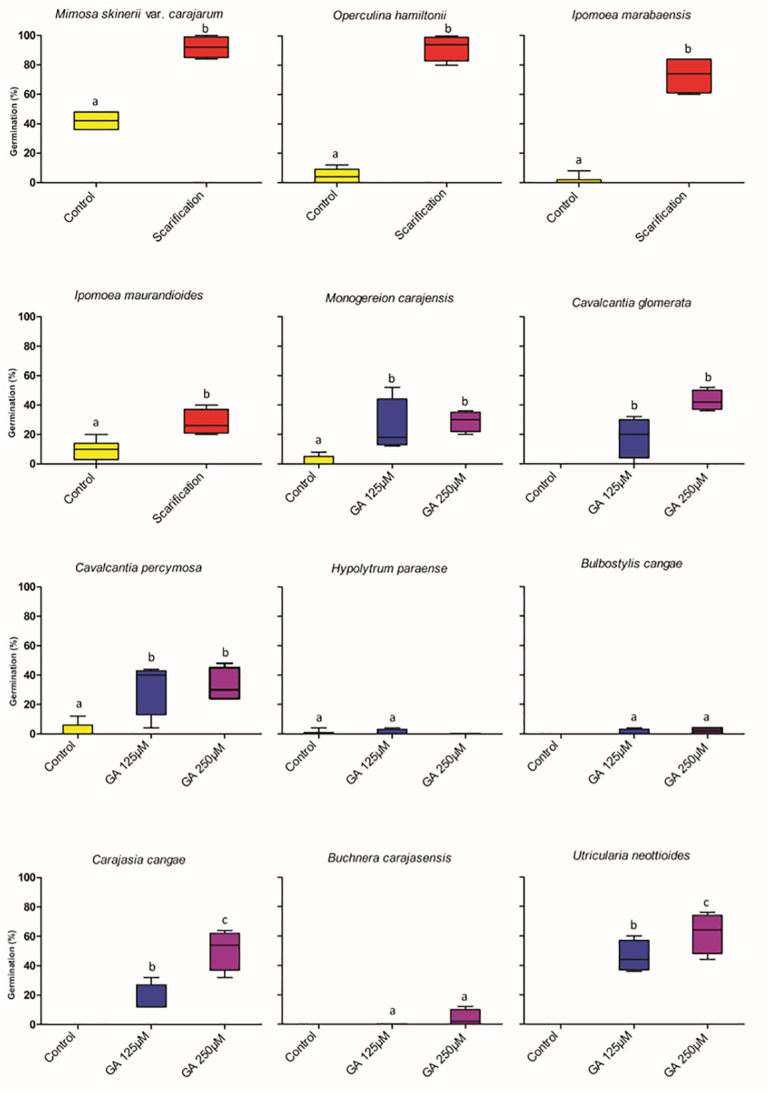
Results of dormancy break experiments in 48 species from ironstone outcrops from eastern Amazon. The boxplots present the germination values showing maximum, minimum and average values. Control of 25°C. G% = percentage of germination. GA = gibberellin.

**FIGURE 4:**
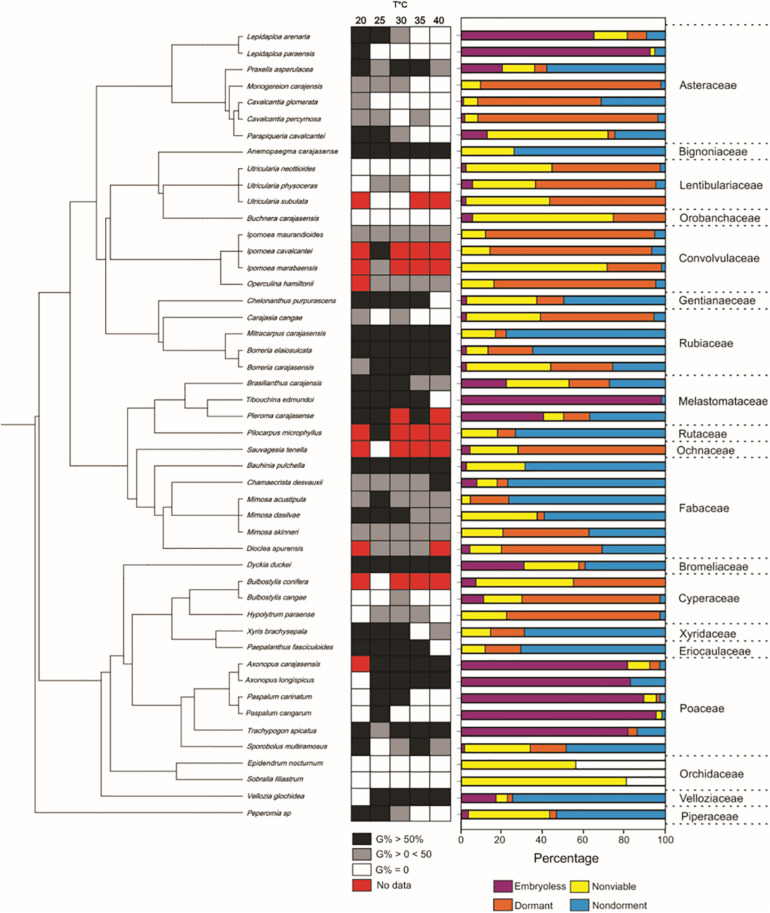
The phylogenetic distribution of seed traits of 48 species from ironstone outcrops in eastern Amazon. G% = germination percentage.

Seventeen species (35.4%) distributed in eight families produced dormant seeds. Among these, five species produced PY seeds and 12 produced PD seeds. Of the 17 species with dormant seeds, only 12 were submitted to dormancy alleviation treatments because of seed shortage (Figure 3). Mechanical scarification was effective in breaking PY in the four species, *Mimosa skinneri* var. *carajarum*, *Ipomoea marabaensis*, *Ipomoea maurandioides* and *Operculina hamiltonii*. For the first three species, scarification resulted in germination percentages greater than 80% of viable seeds, but for *I. maurandioides*, scarification increased germination percentage by 18%. Five species with PD (*Monogereion carajensis*, *Cavalcantia glomerata*, *Cavalcantia percymosa*, *Carajasia cangae* and *Utricularia neottioides*) showed a significant increase in germination percentage when submitted to concentrations of 125 and 250 μM of GA3, and three of them (*C. glomerata, C. cangae* and *U. neottioides*), only germinated under the GA3 treatments, albeit at low percentages (Figure 3). Three species showed no statistical difference in germination percentage among GA3 concentrations, while *Carajasia cangae* and *Utricularia neottioides* showed higher germination percentages when treated with the 250 μM GA3 concentration (Figure 3). Seeds of *Hypolytrum paraensis, Bulbostylis cangae* and *Buchnera carajasensis* did not respond positively to GA3 application. Dormant taxa were concentrated in Convolvulaceae, Cyperaceae, Lentibulariaceae, and in the Eupatorieae (Asteraceae) (Supplementary Material 1 and Figure 4).

### Effects of Seed Storage on Germination

**FIGURE 5:**
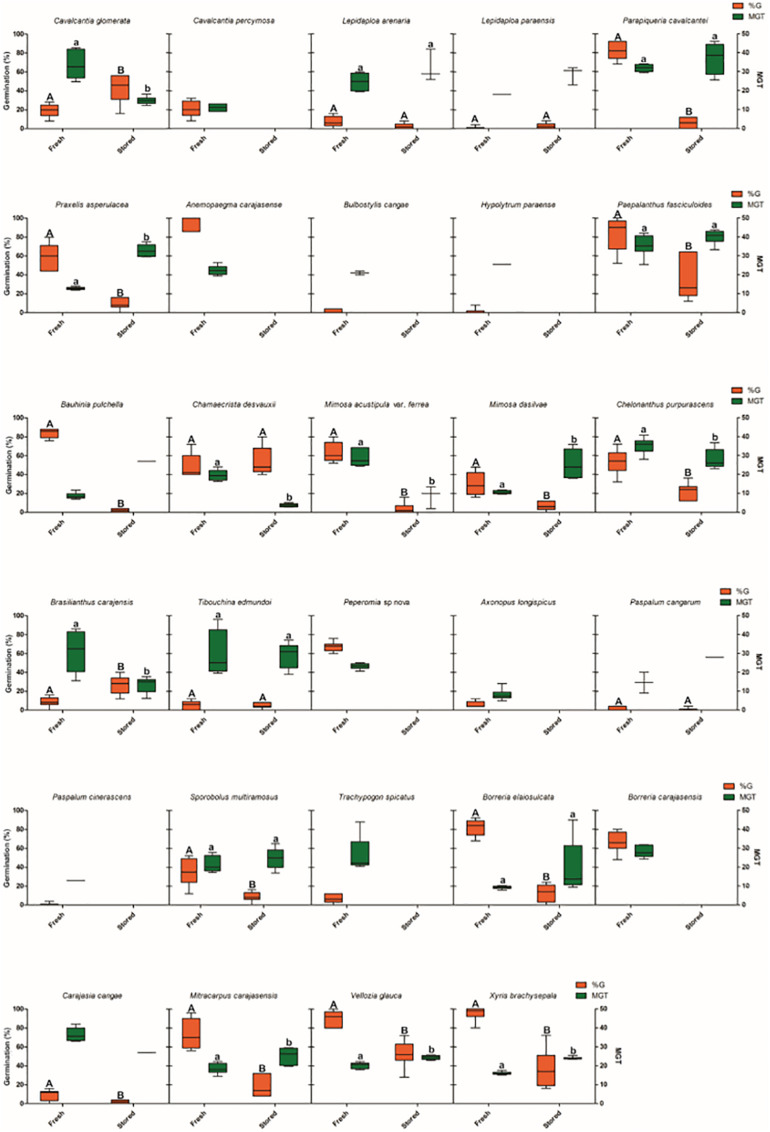
Seed storage effects on germination of 29 species from ironstone outcrops from eastern Amazon. The boxplots present the germination values and mean germination time, showing maximum, minimum and average values. G% = Percentage of germination; MGT = Average germination time (days).

Twenty-two out of twenty-nine species (76%) showed a significant decrease in germination percentage after storage for 24 months (Figure 5). Viability losses ranged from 100% in nine species (*Anemopaegma carajasense*, *Cavalcantia percymosa*, *Bulbostylis cangae*, *Hypolytrum paraense*, *Peperomia* sp., *Axonopus longispicus*, *Paspalum cinerascens*, *Trachypogon spicatus* and *Borreria carajasensis*) to 21% in *Carajasia cangae*. Ten species had an MGT greater than or equal to the MGT for fresh seeds, and only three species showed a decrease in the average germination time.

For five species (*Lepidaploa arenaria*, *Lepidaploa paraenses*, *Chamaecrista desvauxii*, *Tibouchina edmundoi* and *Paspalum cangarum*), we found no difference in the germination percentage after storage for 24 months. Among these species, *Chamaecrista desvauxii* had lower MGT than freshly harvested seeds, the other four species had MGT greater than or equal to the germination rates for freshly harvested seeds (Figure 5). Two species, *Cavalcantia glomerata* and *Brasilianthus carajensis*, showed an increase of 23.3 and 18%, respectively in the germination percentage after storage. These two species also had a lower MGT after storage (Figure 5).

### Integration of Seed Traits With Adult Life-History Traits

**FIGURE 6:**
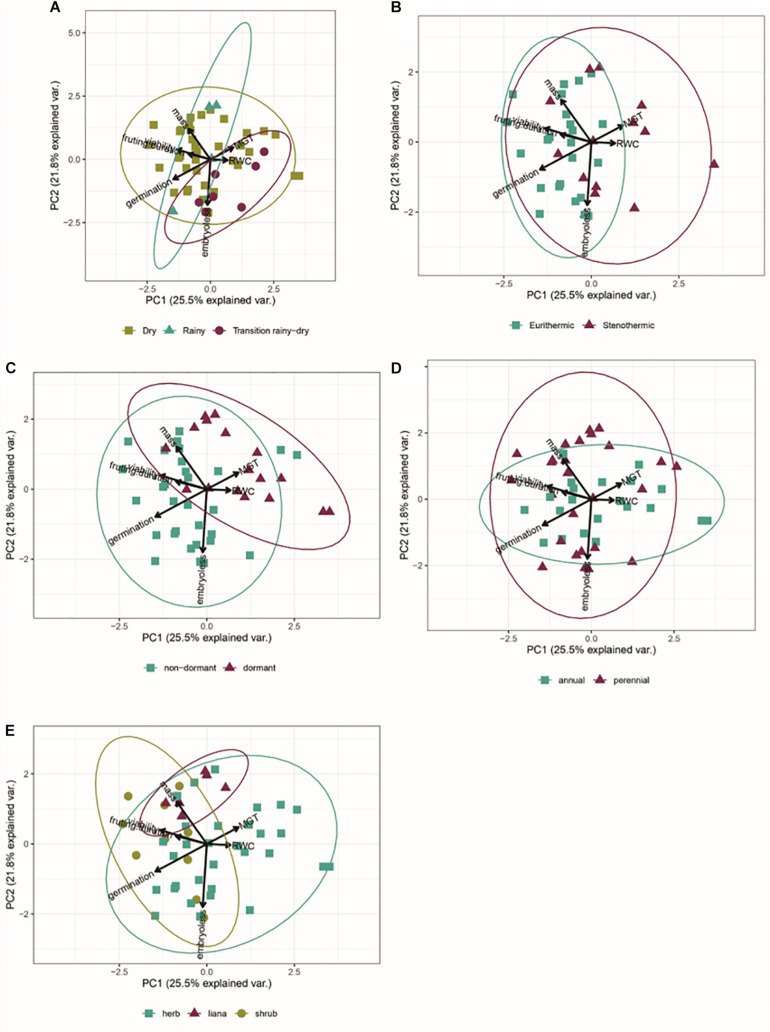
Biplot of the Principal Component Analysis including germination traits of germination percentage, mean germination time, initial viability, seed mass, temperature range, seed water content and fruiting season. **(A)** Dispersal season; **(B)** Germination thermal amplitude; **(C)** Dormancy; **(D)** Life story; **(E)** Growth-form.

We did not find any indication of differences in seed traits among species dispersing seeds during the dry and rainy seasons. Instead, there was high overlap in the multidimensional functional space, suggesting a weak or nonexistent association between germination characteristics and dispersal phenology. However, species dispersing seeds during the rainy-to-dry season transition were associated with a lower percentage of embryoless seeds and higher SWC values (Figure 6A). Stenothermics had seeds with higher SWC and slower germination (Figure 6B). Species with dormant and non-dormant seeds were strongly segregated into the multidimensional functional space (Figure 6C). The functional space of annual and perennial species overlapped, but perennial species were associated with higher percentages of embryoless and higher seed mass seeds (Figure 6D). Finally, we found an overlap between the functional space of herbs and shrubs, but a strong association between lianas and heavier seeds (Figure 6E).

### Phylogenetic Signal

We found low, but significant phylogenetic signal for seed viability, percentage of embryoless seeds and germination percentage (Table 2). We found a relatively strong phylogenetic signal for seed mass. The number of evolutionary transitions observed for thermal niche breadth was not different than expected under the null model (Table 2). The number of evolutionary transitions observed for dormancy was lower than expected by the null model (Table 2). Fruiting season and MGT was not significantly related to phylogenetic affinity (Table 3).

**TABLE 2:** Result of Blomberg’s K test for phylogenetic signal in seven seed functional traits highlighting in bold the traits with significant values.

**Trait**	***K***	***P* values**
Germination percentage	0.433	**0.009**
Viability	0.516	**0.002**
Mean germination time	0.151	0.937
Seed water content	0.320	0.227
Seed mass	0.860	**0.000**
Embryoless	0.607	**0.000**
Fruiting season	0.326	0.216

**TABLE 3:** Result of Maddison and Slatkin (1991) method for phylogenetic signal in the temperature range and dormancy categorical traits, highlighting in bold the traits with significant values.

**Trait**	**Number of trait states**	**Transitions observed**	**Median transitions null model (min–max)**	***P* values**
Dormancy	2	10	14 (7–17)	**0.0109**
Temperature range	2	10	12 (5–14)	0.2031

## Discussion

Ironstone outcrops represent harsh environments under the influence of strong abiotic filters that include high soil temperatures, shallow, metal-rich and nutrient-impoverished soils that restricts establishment by many generalist species, but create conditions for the survival of edaphic specialists (Rajakaruna, 2018; Zappi et al., 2019; Corlett and Tomlinson, 2020). Mining activities pose significant threats for these edaphic endemics, while create opportunities for seed-based restoration strategies. Despite the strong environmental filters at the Amazon *cangas*, we found a broad interspecific variation in most seed traits. This result agrees with the idea that environmental filtering does not necessarily result in trait convergence (Gerhold et al., 2015). We have also found that, in line with previous studies (Moles et al., 2005; Dayrell et al., 2017), dormancy and seed mass were the only traits strongly related to phylogeny, suggesting that phylogenetic relatedness may not be a good predictor of regeneration from seeds in *cangas*, as found for other ecosystems (Barak et al., 2018). This result is in contrast to previous studies showing significant phylogenetic signals in many seed and germination traits (e.g., Arène et al., 2017; Wyse and Dickie, 2017; Gioria et al., 2020). Therefore, phylogenetic relatedness may not be the best way to accurately predict restoration-relevant germination traits in Amazon ironstone outcrops as found for rocky outcrop vegetation elsewhere (Dayrell et al., 2017).

We found little potential for propagation by seeds in some species which have been recommended for restoration of Amazon *cangas* (Giannini et al., 2016). *Tibouchina edmundoi*, both *Lepidaploa* species, and most of our Poaceae species have embryoless seeds percentages >70%. Using these species in restoration either requires increasing seed procurement to compensate for the extremely high number of embryoless seeds or relying on vegetative propagation, with consequential low genetic diversity. Estimates of emergence rates may also be overestimated for those species, and corrections on the potential number of emerged seedlings needs to address the percentages of empty seeds.

We found large interspecific variation in the temperature niche breadth among the studied species. *Canga* plateaus often reach high temperatures (Carmo et al., 2012) and data loggers in Carajas rock outcrops show temperatures of 60°C during the hottest hours of the day (Marino, F., Unpublished data). However, only four species, referred as “ironstone lovers” (Nunes et al., 2015), showed optimum germination at high temperatures of 35–40°C. This indicates that other selective pressures or niche conservatism shape thermal niche breadth in edaphic specialists. Finding suitable recipient sites for species translocation is challenging, especially for the stenothermic species, which have narrower germination niches, and for orchids due to their dependence on mycorrhizae for establishment (Dearnaley, 2007). Translocation operations are currently on their way in Carajás Range, and our study will be useful for selecting recipient sites (Commander et al., 2018).

The overwhelming majority of species showed SWC values lower than 15%, with low interspecific variation, and non-significant phylogenetic signal. *Ex situ* seed storage capacity depends on seed desiccation tolerance, which in turn, can be inferred from SWC (Li and Pritchard, 2009; Wyse and Dickie, 2017). Orthodox seeds (desiccation-tolerant seeds) often have lower SWC values, while recalcitrant seeds (desiccation-sensitive seeds) have higher SWC values and, usually lose viability under *ex situ* storage conditions (Berjak and Pammenter, 2008). However, caution is needed when inferring storage capacity using SWC as a proxy of desiccation tolerance. Our data suggest that *ex situ* conservation is possible for many native species from ironstone outcrops as found for edaphic specialists in analogous metal-rich grasslands in Congo (Godefroid et al., 2020), but specific protocols of *ex situ* seed storage under controlled conditions need to be developed for each species. Currently, accessions of four species (*Mimosa skinneri* var. *carajarum*, *Monogereion carajensis*, *Axonopus carajasensis* and *Ipomoea maurandioides*) have been deposited in the *ex situ* seed bank collections at the Rio de Janeiro Botanic Garden, aiming at new collections new accessions will also be deposited in Embrapa genetic resources and biotechnology, thereby contributing to the targets of the Convention on Biological Diversity (Teixido et al., 2017).

For the 29 species that we stored seeds for a period of 24 months, 13 species (44.8%) showed decreases in germination percentage and nine (32.1%) had null germination after storage. This result suggests these species do not tolerate storage for long periods of time, or that the storage conditions are not suitable for these species (Godefroid et al., 2020). In the absence of reliable information on different methods for storage conditions, our results indicate that seeds of these species should be used as soon as possible after collection to avoid viability loss (Miller et al., 2017). For five species we found no difference in germination percentage between stored and fresh seeds, indicating that these species tolerate storage for periods of up to 24 months at room temperature, and can be stored for conservation programs without viability loss. In turn, seeds of *Cavalcantia glomerata* and *Brasilianthus carajensis* showed 23.3 and 18% increase in germination, respectively, after storage. For these species, after ripening can be a successful strategy for restoration via direct seeding (Baskin and Baskin, 2020).

Nearly 35% of our species produced dormant seeds. Under natural conditions, seed dormancy confers adaptive value by helping spreading germination over time and synchronizing germination timing to periods when seedling establishment is maximized and (Baskin and Baskin, 2014). However, dormancy also prevents homogeneous sapling production in the greenhouse and seedling emergence under field conditions. Dormant seeds on soil seed banks may experience higher mortality due to physiological aging and the action of predators and the action of microorganisms (Long et al., 2015), and therefore, for ecological restoration programs alleviating dormancy is needed either to produce seedlings or to accelerate emergence during seed sowing (Turner et al., 2013; Miller et al., 2017). We show that mechanical scarification was effective in overcoming all PY species, and that GA3 alleviated PD in many species. Therefore, GA3 application can be used to optimize sapling production in greenhouses for these species. Nevertheless, seeds of *Hypolytrum paraensis, Bulbostylis cangae* and *Buchnera carajasensis* did not germinate even under the GA3 application, calling for additional methods to alleviate PD.

We found a strong overlap in seed traits among species fruiting during the rainy and dry seasons. In seasonal environments, natural selection should favor strategies that fine tune establishment with optimum environmental conditions (Baskin and Baskin, 2014), and a co-variation in fruiting phenology and germination strategies is expected (Escobar et al., 2018). Interestingly, the eight species fruiting at the rainy-to-dry season transition were more associated with a higher percentage of embryoless seeds and consequently lower SWC values. Three out of these eight species produce dormant seeds, which should be alleviated during the dry season, and may help synchronize germination with the onset of the next rainy season (Baskin and Baskin, 2014). Dormancy is not a pre-requisite for maintaining seed longevity in the soil (Thompson et al., 2003), so it is possible that the lower SWC values in these eight species increases desiccation-tolerance during the dry winter.

The weak segregation between germination traits in annual and perennial species was also surprising. Annual species are expected to allocate more resources to seeds (Vico et al., 2016), and to produce more dormant seeds since germination during unfavorable conditions for seedling establishment would result in strong detrimental consequences for their populations (Baskin and Baskin, 2014). Our data indicate that perennial species produce higher percentages of embryoless seeds, albeit differences were not remarkable. These results suggest that even annual species which fully rely on seeds to regenerate may be experiencing some level of resource (Fujita et al., 2014) or pollination limitation (Rosbakh et al., 2018) that constrains the production of viable seeds (see also Dayrell et al., 2017). Altogether, the weak association between germination traits and life-history traits indicate that no particular plant functional type requires specific methods for seed-based translocations. Exceptions were the lianas which showed relatively larger seeds compared to the other growth-forms.

Mining activities pose significant threats to *canga* endemics (Zappi et al., 2019), and have negative repercussions that extend to areas far beyond operational lease boundaries (Sonter et al., 2017). Extensive land-use changes result from an increasing global demand for steel (Sonter et al., 2014) suggest an uncertain future for *canga* endemics (Fernandes et al., 2018). Our study is part of a Biodiversity Conservation Plan for the *canga* endemics that intends to contribute to both *in situ* and *ex situ* conservation programs, including to complete the seed germination, dormancy-breaking, and seed storage protocols for all the 38 edaphic endemics and a considerable number of the *canga* community in the coming years. Our study on seed and germination functional traits for 48 species from Amazon *cangas* provides support to restorationists and conservation biologists to better manage seed sourcing, use, storage and enhancement techniques with expected reduced costs and increased seedling establishment success. Testing whether the results found here would apply to other systems under opencast iron-ore mining will be important to determine the generality of our results.

## Data Availability Statement

The raw data supporting the conclusions of this article will be made available by the authors, without undue reservation.

## Author Contributions

MZ, AD, AC, TF, FSa, and FSi conceived the idea. MW and FSa did the field work. MZ conducted the experiments. MZ and RD run statistical analyses. MZ, RD, and FSi wrote the first version of the manuscript. All authors have contributed to the article and approved the submitted version.

## Conflict of Interest

The research was funded by VALE. AD, TF, AC, and FSa are employees of VALE. The remaining authors declare that the research was conducted in the absence of any commercial or financial relationships that could be construed as a potential conflict of interest.
