# Supplementary material for: Seed Functional Traits Provide Support for Ecological Restoration and ex situ Conservation in the Threatened Amazon Ironstone Outcrop Flora
Source: Front Plant Sci. 2020 Dec 8;11:599496. doi: 10.3389/fpls.2020.599496 (PMC7793850; doi:10.3389/fpls.2020.599496)
Supplement: Supplementary file 2 [file Data_Sheet_1.PDF]

We used the Maddison and Slatkin (1991) method to assess the existence of phylogenetic signal in the dormancy categorical trait. This method compares the minimum number of trait-state transitions across a phylogenetic tree with a null model (100,000 randomizations), in which the trait states were randomized in the tips of the tree. If related species have similar trait states, the number of evolutionary transitions observed will be lower than expected based on the null model (Maddison & Slatkin 1991).

Analysis of phylogenetic signal in dormancy across 48 species and temperature range across 39 species of ironstone outcrops. The test compares the number of evolutionary transitions observed with a null model (100,000 randomizations).

Trait	Number of trait states	Transitions observed	Median transitions null model (min – max)	*P*
Dormancy	2	10	14 (7 – 17)	**0.0109**
Temperature range	2	10	12 (5 – 14)	0.2031

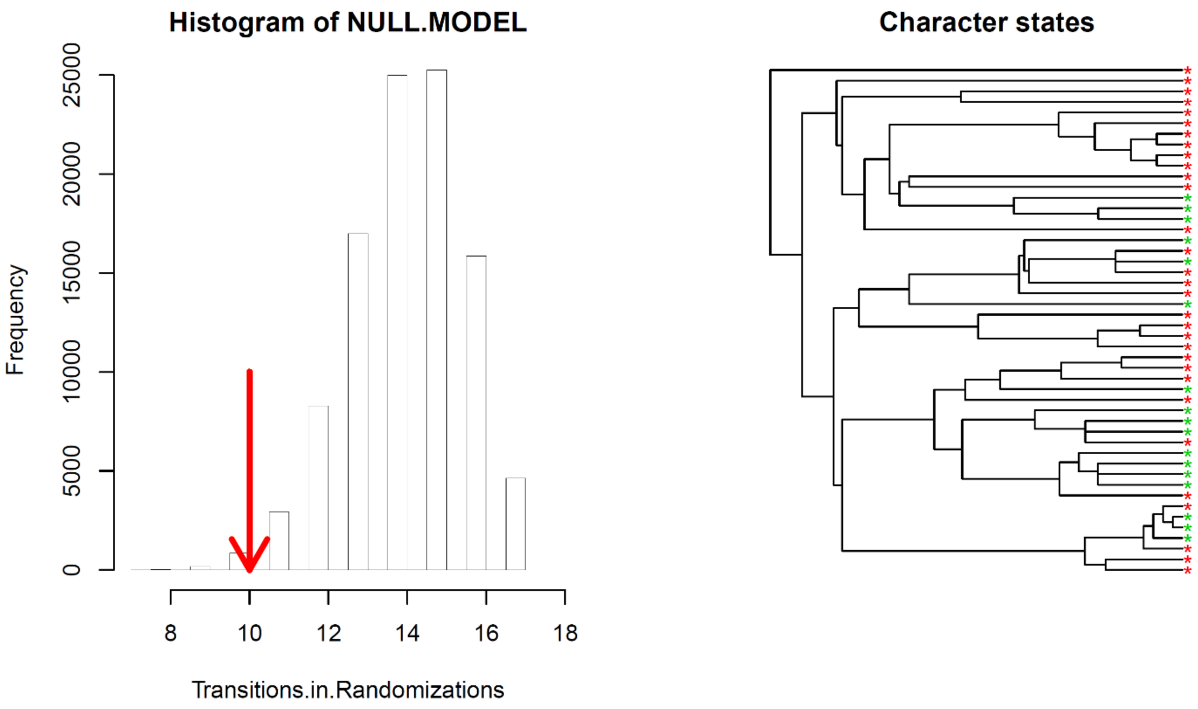

Figures showing the result of Maddison and Slatkin (1999) method for calculating phylogenetic signal in dormancy. Histogram of null model and the observed evolutionary transitions (red arrow); and on the right the phylogenetic tree different trait states are shown in different colours. Red = nondormant; Green = dormant.

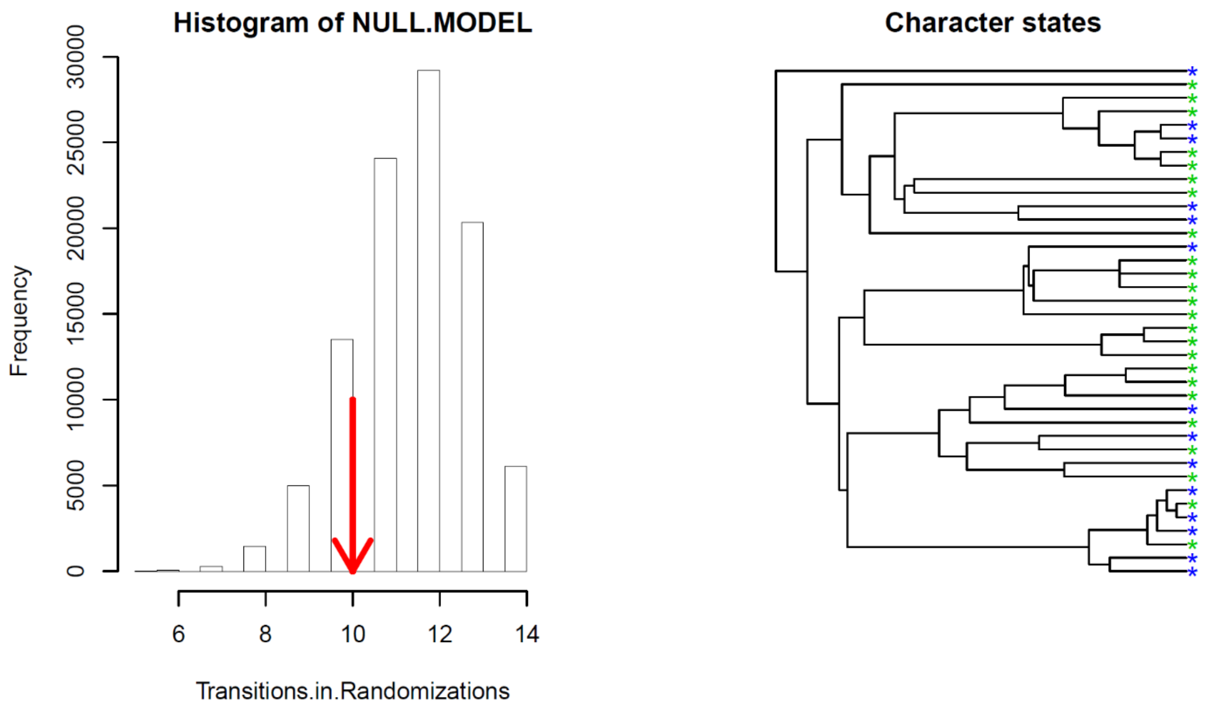

Figures showing the result of Maddison and Slatkin (1999) method for calculating phylogenetic signal in temperature range. Histogram of null model and the observed evolutionary transitions (red arrow); and on the right the phylogenetic tree different trait states are shown in different colours. Green = eurithermic; blue = stenothermic.
